# Complete genome sequence of the unique *Rhizobium johnstonii* strain NaPi

**DOI:** 10.1128/mra.01202-24

**Published:** 2025-02-20

**Authors:** Evgenii A. Kirichek, Alexey M. Afonin, Pyotr G. Kusakin, Viktor E. Tsyganov

**Affiliations:** 1 All-Russia Research Institute for Agricultural Microbiology (ARRIAM), Saint Petersburg, Russia; DOE Joint Genome Institute, Berkeley, California, USA

**Keywords:** legume-rhizobial symbiosis, symbiotic mutants, nodule

## Abstract

In contrast to *Rhizobium johnstonii* strain 3841, *R. johnstonii* strain NaPi is able to form large pink nodules on the roots of pea (*Pisum sativum* L.) mutants in the gene *Sym40*. The genetic determinants underlying such efficiency have not been discovered yet. In this study, we report the complete genome sequence of the strain NaPi.

## ANNOUNCEMENT

**Fig 1:**
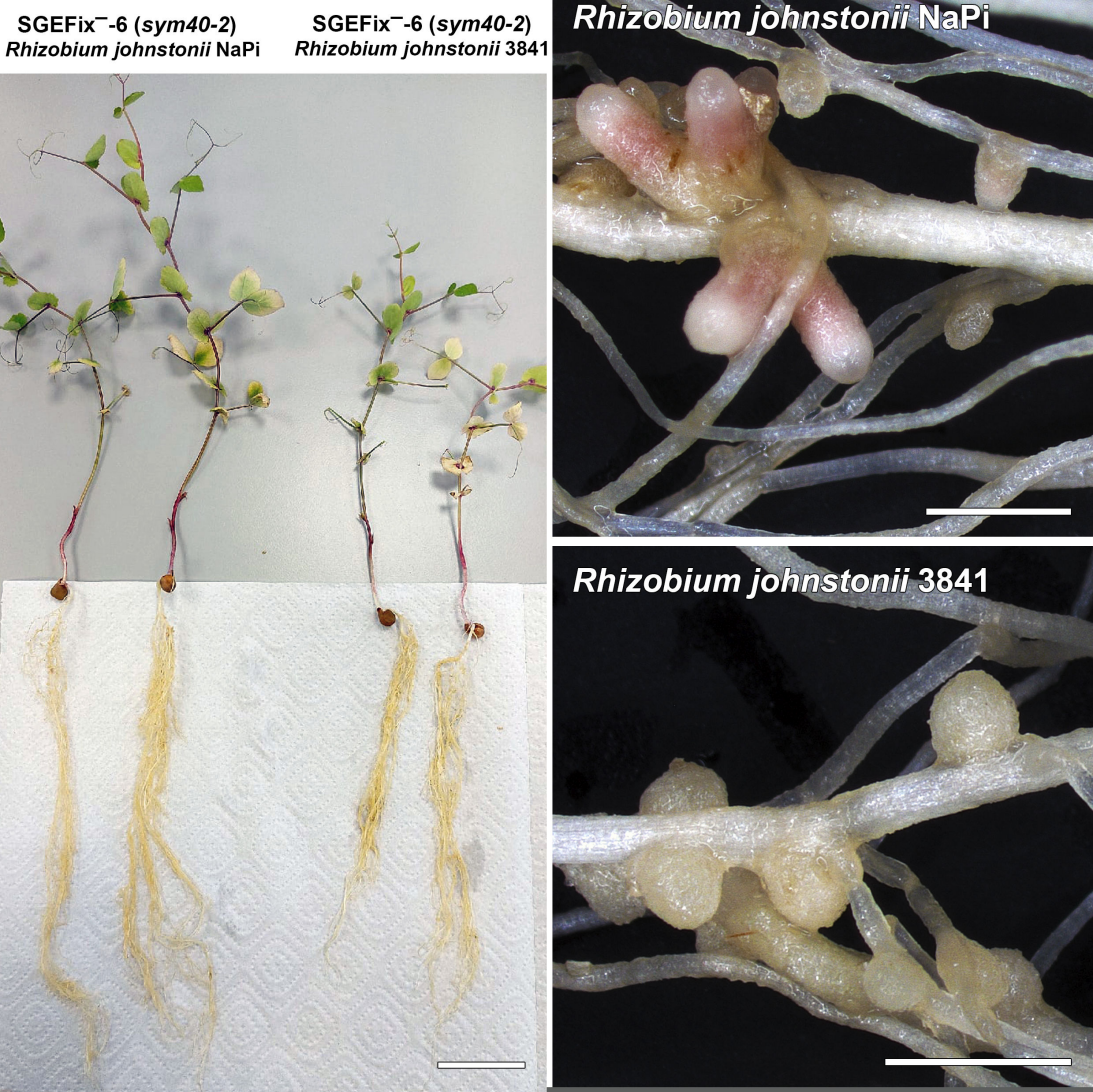
SGEFix^–^-6 (*sym40-2*) pea plants (left) and corresponding nodules (right) after inoculation with *Rhizobium johnstonii* NaPi or *Rhizobium johnstonii* 3841. Bars indicate 2 cm for plant photos or 2 mm for nodule photos.

Active use of symbiotic nitrogen fixation leads to a reduction in the application of nitrogen fertilizers. For this purpose, it is necessary to identify and study effective rhizobia strains. *Rhizobium johnstonii* strain 3841 (1) is widely used in studies of legume-rhizobial symbiosis (2–4). *R. johnstonii* strain NaPi has been shown to form large pink nodules on the roots of *sym40* pea symbiotic mutants (Fig. 1), compared to the typically smaller white nodules associated with the *sym40* genotype (5). The pea *Sym40* gene is an ortholog of the *Medicago truncatula EFD* gene (6) encoding a negative regulator of cytokinin response (7).

During laboratory experiments, a spontaneous pink nodule was found on the pea plant mutant SGEFix^–^−1 (*sym40-1*), which was grown in sterile vermiculite and inoculated with *R. johnstonii* strain 3841. *Rhizobium* strain was isolated from that pink nodule on plates containing tryptone-yeast extract (TY) medium, and incubated for a week at 28°C according to Vincent (8). Strain was named NaPi and preserved in TY medium with 40% glycerol at −80°C. For DNA isolation, strain was revived on a solid TY medium and incubated for 72 h at 28°C. DNA was isolated using the phenol-chloroform method (9) without size selection.

Long-read sequencing was performed using a MinION with flow cell R.9.4.1; the SQK-LSK109 kit with EXP-NBD104 and EXP-NBD114 was used to prepare the library according to the manufacturer’s instructions skipping the DNA-shearing step. The reads were base called and demultiplexed using Guppy v.3.3.0 (high accuracy model) with default parameters. 92,680 nanopore reads with a total length of 1,032,198,719 bp, *N*_50_ = 20,059, and estimated coverage of 133× were obtained. The *de novo* assembler Flye (10) v.2.6 was used to assemble raw reads. The assembly was corrected four times using Racon (11) v.1.3.2 (with -m 8 x -6 g -8 w 500 options), and polished with medaka (12) v.0.10.0 using long reads.

Short-read sequencing was carried out on an Illumina HiSeq X Ten with the TruSeq DNA PCR-Free kit according to the manufacturer’s recommendations. In total, 4,415,831 sequence reads of 2 × 150 bp were generated. The reads were quality trimmed and adapters were removed as described (13); the expected coverage was about 166×. The short reads were used to polish the assembled genome using Pilon (14) v.1.22. Default parameters were used for all listed software unless otherwise noted.

The genome consists of seven assembled fragments, including one chromosome and six plasmids with a total length of 7,753,359 bp, assembly *N*_50_ = 5,059,001, and an average GC content of 60.86%. Complete genome was deposited at GenBank and annotated using the NCBI Prokaryotic Genome Annotation Pipeline (PGAP) (15) v.6.8. Overall, 7,440 CDSs and 65 RNAs were annotated.

The circularity of all assembled fragments was verified by mapping the long reads to the assembled fragments using Minimap2 (16), with the map-ont mapping mode, and inspecting the coverage uniformity. The chromosome was rotated, the *dnaA* gene was placed at the start of the sequence; for plasmids, a *repABC* operon was placed at the start of the sequence.

## Data Availability

All data are available in the NCBI database under the BioProject accession number PRJNA1177650. The assembly accession numbers are CP172584.1 to CP172590.1. The raw Illumina data can be found under number SRR31112948, and the ﬁles from the MinION runs deposited under accession number SRR31130023. This announcement describes the ﬁrst version of the genome assembly.
